# Pathologic complete response and survival in early-stage HER2-positive breast cancer patients treated with or without anthracyclines

**DOI:** 10.3332/ecancer.2026.2118

**Published:** 2026-05-06

**Authors:** Francisco Acevedo, Benjamín Walbaum, Lidia Medina, Maritza Abud, Roger Gejman, Pablo Zoroquiain, Francisco Domínguez, Mauricio Camus, Catalina Vargas, Marisel Navarro, Constanza Pinto, Catalina Muñoz, Manuel Manzor, César Sánchez

**Affiliations:** 1 Department of Hematology and Oncology, School of Medicine, Pontificia Universidad Católica de Chile, Santiago 8320165, Chile; 2 “Nuestra Señora de la Esperanza” Cancer Center, UC Christus Health Network, Pontificia Universidad Católica de Chile, Santiago 8320165, Chile; 3 Department of Pathology, School of Medicine, Pontificia Universidad Católica de Chile, Santiago 8320165, Chile; 4 Department of Surgery, School of Medicine, Pontificia Universidad Católica de Chile, Santiago 8320165, Chile; 5 Dr. Sótero del Rio Hospital and Healthcare Complex, Santiago 8207257, Chile; 6 Department of Biochemistry, School of Biology, Pontificia Universidad Católica de Chile, Santiago 8320165, Chile

**Keywords:** HER2-positive breast cancer, anthracyclines, neoadjuvant chemotherapy, trastuzumab, pathologic complete response

## Abstract

The development of novel human epidermal growth factor receptor type-2 (HER2)-targeted therapies for HER2-positive breast cancer (HER2+ breast cancer (BC)) in recent decades has called for a reassessment of the benefit of including anthracyclines in neoadjuvant regimens, given their association with cardiotoxicity and secondary malignancies. Our study assessed the value of adding anthracyclines in a real-world cohort of early-stage HER2+ BC patients treated with trastuzumab with or without pertuzumab. We retrospectively evaluated 446 patients with early-stage HER2+ BC treated with neoadjuvant chemotherapy at two Chilean centers between 2010 and 2023. Patients received trastuzumab alone or trastuzumab plus pertuzumab, with anthracycline-containing or anthracycline-free regimens. Our primary endpoint was pathological complete response (pCR; ypT0/is ypN0). Secondary endpoints included invasive disease-free survival (iDFS) and overall survival (OS). Multivariate models assessed the predictive role of anthracyclines and pathological biomarkers (ER status, Ki67, HER2 amplification). Elevated Ki67, low ER expression and increased HER2 amplification were independent predictors of pCR. The addition of anthracyclines did not significantly improve pCR rates, even among patients treated with single HER2 targeted therapy (trastuzumab alone). With a median follow-up of 47 months, anthracyclines had no impact on iDFS (log-rank p = 0.19) or OS (p = 0.96). Subgroup and interaction analyses confirmed no benefit in other biomarker-defined populations. Overall, anthracyclines did not improve response to treatment or survival in HER2+ BC, even without dual HER2 blockade. These findings support anthracycline-free regimens, even in health systems with limited access to dual HER2-blockade with pertuzumab.

## Background

Anthracyclines are a class of cytotoxic drugs with potent antineoplastic activity and are widely used against several malignancies, including breast cancer (BC). Although anthracyclines act primarily by DNA intercalation and topoisomerase II inhibition, they also generate reactive oxygen species, further enhancing their cytotoxic activity [1]. For decades, the combination of anthracyclines and taxanes has been the cornerstone of systemic chemotherapy treatments in early-stage BC [2]. Prior to the development and widespread adoption of human epidermal growth factor receptor type-2 (HER2)-targeted therapies (such as trastuzumab), anthracyclines displayed preferential benefits for HER2+BC and therefore were included as a key component of chemotherapy regimens for this subtype [3]. However, their use has always been accompanied by concerns regarding severe acute and long-term toxicities, including cardiotoxicity and the potential risk of secondary leukemia [4–6].

The development of novel HER2-targeted therapies in recent years, along with alternative chemotherapy regimens, has sparked the debate on the necessity of including anthracyclines in modern BC treatments [7, 8]. In 2011, a pivotal study by Slamon *et al* [9] compared the efficacy and safety of adjuvant docetaxel, carboplatin plus trastuzumab versus doxorubicin, cyclophosphamide, docetaxel (AC-T) plus trastuzumab and demonstrated that similar disease-free survival (DFS) and overall survival (OS) levels were accompanied by lower cardiotoxicity. Subsequently, several studies and meta-analyses have confirmed that omitting anthracyclines does not have a significant impact on the efficacy of pertuzumab plus trastuzumab (dual HER2-blockade) treatments in early-stage HER2+BC [9, 10]. The implementation of these strategies has also been associated with a significant reduction in severe adverse events and toxicities [3]. Unfortunately, the access to dual HER2-blockade is not universal or affordable for many healthcare systems across the globe [11, 12], and while several studies have shown a lack of benefit by anthracyclines in the presence of dual blockade, only a few have focused on anthracyclines with single HER2-targeted therapy using trastuzumab alone.

Achieving a pathological complete response (pCR) after treatment is a reliable surrogate marker for long-term survival in HER2+ BC undergoing neoadjuvant therapy [13, 14]. Studies demonstrate that pCR correlates with a reduced risk of recurrence and improved OS, also reflecting tumour chemosensitivity [15, 16]. Consequently, studies have sought to identify predictive biomarkers of pCR to stratify patients according to their likelihood of therapy response [15–17].

As explained, treatment regimens that omit anthracyclines can reduce treatment-related toxicity while maintaining their efficacy. However, most of these regimens include dual HER2 blockade [18, 19]. To date, there are no consistent data on the use of anthracyclines in settings with limited access to dual blockade.

Our study evaluated the effect of anthracyclines on pCR rates and survival in a cohort of HER2+ BC patients, with or without dual HER2-blockade. We also performed an exploratory analysis to determine the role of HER2 amplification and ER expression levels as biomarkers that could identify patients who benefited from anthracycline-containing regimens.

## Methods

### Study patients

Adult (≥18 year-old) women diagnosed with HER2+ BC who received neoadjuvant chemotherapy (NAC) between 2010 and 2023 were retrospectively included into the study. Participants were treated at two cancer centers in Santiago, Chile: the Pontificia Universidad Católica de Chile, a tertiary academic hospital, and Dr. Sótero del Río healthcare complex, a public oncology center. A study protocol was approved by the institutional ethics committees at both institutions. All patient data were anonymised to protect patients` privacy and to ensure confidentiality.

### Eligibility criteria

Patients with histologically confirmed invasive HER2+ BC, completed NAC followed by surgical resection and complete clinical, biomarker, treatment and pathological outcome data were eligible. Positivity for HER2 (HER2+) was defined according to the ASCO/CAP 2018 guidelines [20]. requiring an IHC score: 3+ or an IHC score: 2+ with confirmed HER2 amplification by *in situ* hybridisation (ISH). Patients with *de novo* metastatic disease or incomplete clinical records were excluded.

### Measurement of biomarkers

HER2 amplification and the HER2/CEP17 ratio were quantified using ISH and analysed both as continuous variables (gene copy number) and as categorical variables based on tertiles (low, medium, high). ER expression was recorded both as a continuous percentage and dichotomously: low (0%–10%) or high (>10%). Additional covariates included the Ki-67, stage and histological grade. Treatment-related variables included exposure to pertuzumab (dual HER2 blockade) and receiving anthracycline-containing regimens.

### Primary and secondary outcomes

Our primary endpoint was pCR, defined as ypT0/isN0, indicating the absence of invasive cancer in the breast tissue and axillary lymph nodes, irrespective of the presence of residual *in situ* carcinoma. Secondary endpoints included invasive disease-free survival (iDFS) and OS. iDFS was defined as the time from definitive surgery to the first documented occurrence of invasive disease recurrence, including local, regional or distant relapse, the development of a second primary invasive cancer or death from any cause. OS was defined as the time from surgery to death from any cause. Patients who did not experience the event of interest were censored at the last follow-up date. Both iDFS and OS were evaluated to assess long-term outcomes in relation to anthracycline use and biomarker-defined patient subgroups.

### Statistical analysis

Descriptive statistics were used to summarise clinical and pathological characteristics. Bivariate comparisons between pCR and non-pCR groups were performed using the chi-square or Fisher’s exact test for categorical variables and Student’s *t*-test or Mann–Whitney *U* test for continuous variables, depending on data distribution. Predictors of pCR were identified via multivariate logistic regression models, incorporating clinical and biomarker variables that displayed significance in univariate analyses. Results were reported as odds ratios (ORs) with 95% confidence intervals (CIs). Survival (OS and iDFS) was analysed using the Kaplan–Meier method. Patients without events were censored at the date of last follow-up. Survival differences between groups were assessed using the log-rank test. The proportional hazards assumption was verified using Schoenfeld residuals. Hazard ratios with 95% CIs were reported.

### Subgroup and interaction analyses

Subgroup analyses were conducted stratifying by HER2 metrics (IHC score, amplification level and HER2/CEP17 ratio), ER status and pertuzumab exposure. Interaction terms between anthracycline exposure and biomarker categories (e.g., HER2 IHC 3+ and anthracycline use, ER high and anthracycline use) were incorporated into logistic regression models to evaluate potential effect modification. Marginal effects were derived using the margins command in Stata to estimate predicted probabilities across interaction strata. All analyses were performed using Stata v.16 (StataCorp, College Station, TX).

## Results

A total of 446 patients with early stage HER2+ BC were included into our study. First, we sought to identify clinical variables associated with pCR in patients treated with or without anthracyclines. Table 1 shows that higher Ki67 (50.0% versus 35.0%; *p* = 0.030 and 40.0% versus 30.0%; *p* = 0.027), lower ER expression (20.0% versus 90.0%; *p* = 0.012 and 0.0% versus 85.5%; *p* < 0.001) and categorised HER2 metrics, including HER2 IHC score (IHC 3+: 58.57% versus 41.43%; *p* = 0.017 and 57.19% versus 42.81%; *p* < 0.001), HER2 amplification level (high copy number tertile: 67.74% versus 32.26%; *p* = 0.006 and 62.04% versus 37.96%; *p* < 0.001) and HER2/CEP17 ratio (high ratio tertile: 68.97% versus 31.03%; *p* < 0.001 and 62.71% vesus 37.29%; *p* < 0.001) were significantly associated with pCR in both groups.

**Figure 1.**
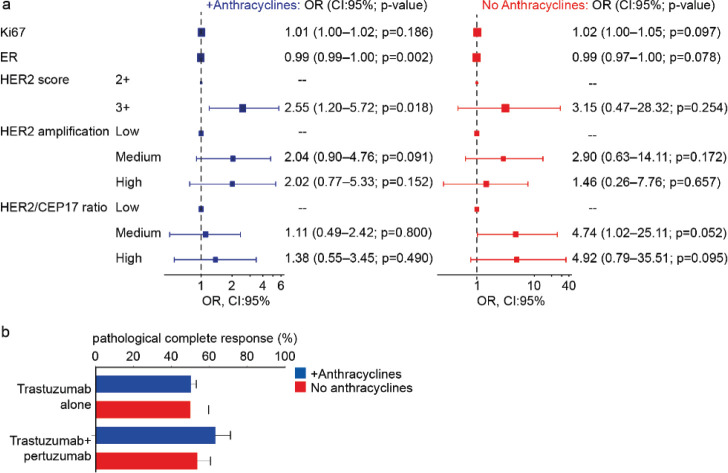
Predictive biomarkers and treatment response with and without anthracyclines in HER2-positive BC. (a) Forest plots showing OR and 95% (CI: 95%) for pCR according to biomarker status, stratified by treatment regimen with anthracyclines (blue, left) or without anthracyclines (red, right). (b) pCR rates (%) in patients receiving trastuzumab alone or dual HER2 blockade with trastuzumab plus pertuzumab, with (blue bars) and without (red bars) anthracyclines. Abbreviations: ER: estrogen receptor, HER2: human epidermal growth factor receptor type 2; CEP17: chromosome enumeration probe-17.

Then, we evaluated the impact of Ki67, ER expression, HER2 metrics and anthracycline exposure on the likelihood of achieving pCR and performed multivariate logistic regression analyses. Left and right panels in Figure 1a shows a comparison of forest plots with or without anthracyclines. Note that most variables displayed similar OR levels (Ki67: 1.01 versus 1.02, high HER2 amplification: 2.02 versus 1.46, high HER2/CEP17 ratio: 1.38 versus 4.92) that did not reach statistical significance when comparing anthracycline-containing (blue) versus anthracycline-free (red) regimens (*p* = 0.186, *p* = 0.097, *p* = 0.152, *p* = 0.657, *p* = 0.490 and *p* = 0.095, respectively), except for HER2 IHC score 3+ where the use of anthracyclines was associated with a higher probability of pCR (OR = 2.55; 95% CI 1.20–5.72; *p* = 0.018). Next, we compared pCR levels in patients that received neoadjuvant therapies that contained trastuzumab alone versus trastuzumab plus pertuzumab, with or without anthracyclines. As shown by Figure 1b, the addition of anthracyclines had no significant effect on absolute pCR levels in patients that received treatments with (63.2% versus 53.7%; *p* = 0.372) or without pertuzumab (50.0% versus 50.3%; *p* = 0.975).

**Figure 2.**
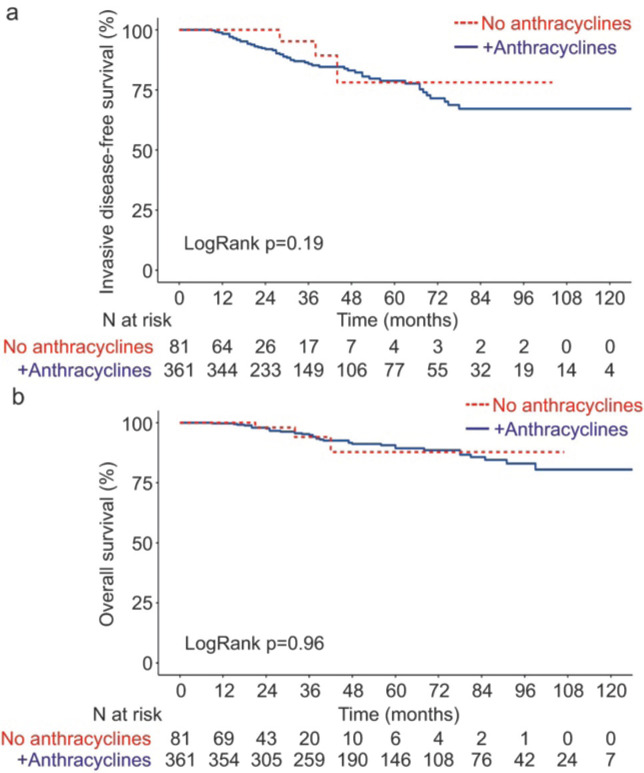
Five-year survival rates in HER2-positive BC patients treated with or without anthracyclines. (a) Kaplan–Meier estimates of iDFS for patients treated with anthracyclines (blue solid line) versus without anthracyclines (red dashed line). LogRank p = 0.19. (b) Kaplan–Meier estimates of OS comparing anthracycline-containing (blue solid line) and anthracycline-free (red dashed line) regimens (LogRank p = 0.96). Number of patients at risk are shown below each plot.

Subsequently, we assessed the effect of anthracyclines on patient survival. Figure 2 shows that using a median follow-up of 47 months, anthracyclines did not have a significant effect in either 5-year iDFS (LogRank *p* = 0.19) or 5-year OS (LogRank *p* = 0.96). Similarly, the addition of pertuzumab did not have a significant effect in 5-year iDFS (LogRank *p* = 0.95) or 5-year OS (LogRank *p* = 0.64).

## Discussion

Consistent with the current literature, our study found that elevated Ki67, low ER expression and HER2 metrics (IHC and amplification) were associated with pCR in patients with HER2+ BC receiving neoadjuvant therapy. Our results also suggest no significant benefit in pCR by the addition of anthracyclines, even without dual HER2-blockade therapies (trastuzumab plus pertuzumab). Moreover, anthracyclines did not affect survival outcomes (iDFS or OS), suggesting a lack of benefit even in resource-constrained settings where the use of more intensified treatments could be considered due to limited access to targeted therapies. Overall, our results suggest that tumour biology, rather than treatment escalation, is a key determinant of response in HER+BC patients.

Previously, the phase III TRAIN-2 trial demonstrated that neoadjuvant anthracycline-free regimens were equally effective versus anthracycline-containing treatments [18]. Additionally, omitting anthracyclines was associated with fewer side effects and toxicity. In recent years, several systematic reviews and meta-analyses confirmed a lack of significant benefit in pCR rates or event-free survival by the addition of anthracyclines in HER2+ BC [9, 21, 22]. These studies also confirmed the association between anthracyclines and an elevated risk of cardiotoxicity. Interestingly, a recent retrospective study by Iwase *et al*. [23] compared neoadjuvant HER2-targeted regimens with or without anthracyclines and found similar pCR rates and OS levels in patients with HER2+ inflammatory BC, a rare and aggressive subtype of BC clinically characterised by rapid progression and early recurrence [24]. In line with these studies, our multivariate analyses confirmed a lack of pCR benefit by anthracyclines after adjusting for Ki67 levels, ER expression and HER2 metrics. Conversely, HER2 amplification remained a significant predictor of pCR in both anthracycline-free and anthracycline-containing subsets. However, the magnitude of association appeared modestly attenuated in the anthracyclines group, suggesting the benefit of HER2-targeted therapy is not potentiated by treatment intensification. On the other hand, interaction analyses found no significance between anthracycline and HER2 or ER expression status, further confirming no anthracycline benefit in any biomarker-defined subgroup.

Importantly, although health policies in Chile guarantee ISH testing for all patients with scores 2+ and 3+ in HER2 IHC, neoadjuvant pertuzumab (and consequently dual HER2 blockade) is not approved in public hospitals, offering a unique real-world setting to examine pCR predictors across varying treatment intensities. As occurs in Chile, several low- and middle-income countries face similar limitations in the access to dual HER2 blockade [11, 12]. As pointed earlier, our results confirm a lack of pCR benefit by the addition of anthracyclines even in patients that received trastuzumab alone. Notably, a previous retrospective study from India that included non-metastatic HER2+ BC treated with neoadjuvant single HER2-targeted therapy (trastuzumab only) reported no significant differences in pCR, DFS or OS between anthracycline-free and anthracycline-containing regimens [25]. Similarly, China approved the use of pertuzumab in 2020. A recent report analysed and compared two trastuzumab-based neoadjuvant regimens with or without anthracyclines in HER2+ BC patients enrolled between 2013 and 2019 [26]. This study did not find significant differences in pCR, EFS or OS by the inclusion of anthracyclines. However, toxicity was significantly lower in the anthracycline-free group.

In summary, given the well-documented risk of cardiotoxicity and the lack of additional benefit in terms of response and survival, our findings support the use of anthracycline-free neoadjuvant treatment regimens in the management of HER2+BC [27]. even in the absence of dual HER2 blockade. Furthermore, these results suggest that biomarker-guided de-escalation in this subset of patients is both feasible and rational [28]. In this regard, HER2 metrics and ER status are robust and readily accessible predictors of treatment response. Therefore, these markers can guide neoadjuvant treatment decisions, especially in settings with limited access to advanced molecular profiling or targeted therapies, such as the Chilean public health system.

Our study has several limitations. First, this was a retrospective, observational study. Therefore, treatment allocation, including the use of anthracyclines and pertuzumab were not randomised and could have been influenced by specific institutional protocols or by the availability of treatment(s), potentially introducing a selection bias. Second, the proportion of patients that did not receive anthracyclines (no anthracyclines subgroup) was relatively small (*n* = 82 versus *n* = 364 with anthracyclines) and, therefore, our findings should be interpreted with caution. Third, although our multivariate analyses were adjusted for known confounders, there were unmeasured variables that could not be ruled out. Fourth, HER2 and ER status were evaluated using IHC and ISH, respectively. Future studies should incorporate more advanced molecular techniques such as RNA sequencing [29] or digital spatial profiling [30] to achieve better predictive accuracy.

## Conclusion

In this real-world cohort, anthracyclines did not modify pCR rates or survival in patients with HER2+ BC, regardless of concomitant treatment with either trastuzumab alone or trastuzumab plus pertuzumab. This finding was consistent across subgroups, including age, disease stage, body mass index, histological grade, ER status and Ki-67 index. In contrast, HER2 metrics (IHC, amplification, HER2/CEP17 ratio) and low ER consistently predicted pCR, confirming the key role of tumour biology in treatment response. In healthcare settings where dual HER2 blockade is still unavailable, our findings indicate no additional therapeutic benefit from anthracyclines, supporting the adoption of biomarker-driven de-escalation strategies to optimise treatment efficacy while minimising toxicity.

## Conflicts of interest

The authors have no relevant financial or non-financial interests to disclose.

## Funding

This work was partially funded by an ‘Agencia Nacional de Investigacion y Desarrollo’ (ANID) grant FONDECYT Iniciacion #11240976 (to FA).

## Ethical approval and consent to participate

This was an observational study. The Scientific and Ethics Committee for Health Sciences at the Pontificia Universidad Catolica de Chile approved the study (approval ID# 221104002). The Scientific and Ethics Committee for Health Sciences at the Pontificia Universidad Catolica de Chile also granted waiver of consent to participate in patients.

## Data availability

The datasets generated during and/or analysed during the current study are not publicly available because some dates could lead to the identification of patients. However, the anonymised data are available from the corresponding author (CS) on reasonable request.

## Author contributions

Francisco Acevedo, Benjamin Walbaum and Cesar Sanchez developed the concept and designed the study. Material preparation, data collection and analysis were performed by Lidia Medina, Maritza Abud, Roger Gejman, Pablo Zoroquiain, Francisco Dominguez, Mauricio Camus, Catalina Vargas, Marisel Navarro, Constanza Pinto, Catalina Muñoz and Manuel Manzor. The first draft of the manuscript was written by Francisco Acevedo, Benjamin Walbaum and Cesar Sanchez and all authors commented on previous versions of the manuscript. All authors read and approved the final manuscript.

## Figures and Tables

**Table 1.** Basic patient characteristics and pCR associated variables stratified by anthracycline use.

Variable; units	All patients (n = 446)	No anthracyclines (n = 82)			+Anthracyclines (n = 364)		
		No pCR	pCR	p-value	No pCR	pCR	p-value
Median age; year (range)	51.4 (24.4–84.0)	53.3 (32.9–79.4)	53.2 (36.0–83.9)	0.993	50.0 (26.2–79.1)	52.3 (24.4–78.8)	0.134
Median BMI; kg/m^2^ (range)	28.2 (18.2–50.2)	26.1 (18.2–41.5)	29.0 (18.9–40.6)	0.205	28.2 (18.7–49.9)	28.1 (18.5–50.1)	0.92
Type of institution; *n* (%)							
Public	313 (70.18)	12 (57.14)	9 (42.86)	0.317	144 (49.32)	148 (50.68)	0.459
Private	133 (29.82)	27 (44.26)	34 (55.74)		32 (44.44)	40 (55.56)	
Stage				0.385			0.077
I–II	219 (58.4)	24 (43.6)	31 (56.36)		87 (53.05)	77 (46.95)	
III	156 (41.6)	9 (56.4)	7 (43.75)		60 (42.86)	80 (57.14)	
Median Ki67; % (range)	40 (1.0–97.0)	35.0 (5.0–90.0)	50.0 (15.0–95.0)	0.030	30.0 (2.0–97.0)	40.0 (1.0–90.0)	0.027
Histological grade; *n* (%)				0.145			0.360
1–2	61 (30.8)	11 (57.89)	8 (42.11)		22 (52.38)	20 (47.62)	
3	137 (69.2)	12 (34.29)	23 (65.71)		49 (48.04)	53 (51.96)	
Median ER expression (range)	40 (0–100)	90.0 (0–100)	20.0 (0–100)	0.012	85.5 (0–100)	0.0 (0–100)	<0.001
ER by categories; *n* (%)				0.341			<0.001
ER-negative (ER = 0%)	166 (37.2)	10 (47.62)	11 (52.38)		50 (34.48)	95 (65.52)	
ER low (ER 1%–10%)	26 (5.8)	0 (0)	8 (100)		8 (44.44)	10 (55.56)	
ER high (ER>10%)	254 (57.0)	29 (54.72)	24 (45.28)		118 (58.71)	83 (41.29)	
HER2 score by IHC; *n* (%)				0.017			<0.001
2+	62 (14.2)	9 (81.82)	2 (18.18)		40 (78.43)	11 (21.57)	
3+	376 (85.8)	29 (41.43)	41 (58.57)		131 (42.81)	175 (57.19)	
Median HER2/CEP17 ratio (range)	5.10 (1.3–67.9)	4.00 (1.3–30.6)	5.90 (2.5–67.9)	0.181	4.58 (1.4–50.5)	5.55 (1.3–20.7)	0.061
HER2/CEP17 ratio tertiles; *n* (%)				<0.001			<0.001
Low (<4)	143 (32.5)	18 (78.26)	5 (21.74)		79 (65.83)	41 (34.17)	
Medium (≥4–<6.18)	150 (34.1)	12 (40.00)	18 (60.00)		53 (44.17)	67 (55.83)	
High (≥6.18)	147 (33.4)	9 (31.03)	20 (68.97)		44 (37.29)	74 (62.71)	
HER2 amplification tertiles; *n* (%)				0.006			<0.001
Low (>8.75)	140 (32.9)	18 (69.23)	8 (30.77)		76 (66.67)	38 (33.33)	
Medium (8.75–12.9)	147 (34.5)	7 (35.00)	13 (65.00)		51 (40.16)	76 (59.84)	
High (>12.9)	139 (32.6)	10 (32.26)	21 (67.74)		41 (37.96)	67 (62.04)	
Pertuzumab use; *n* (%)				0.754			0.132
Yes	92 (20.6)	25 (46.3)	29 (53.7)		14 (36.8)	24 (63.2)	
No	354 (79.4)	14 (50)	14 (50)		162 (49.7)	164 (50.3)	
